# Neuroprotective Role of Selected Antioxidant Agents in Preventing Cisplatin-Induced Damage of Human Neurons In Vitro

**DOI:** 10.1007/s10571-019-00667-7

**Published:** 2019-03-14

**Authors:** Jelena Popović, Andrijana Klajn, Tatjana Paunesku, Qing Ma, Si Chen, Barry Lai, Milena Stevanović, Gayle E. Woloschak

**Affiliations:** 1 Institute of Molecular Genetics and Genetic Engineering, University of Belgrade, Belgrade, 11010 Serbia; 2 Feinberg School of Medicine, Department of Radiation Oncology, Northwestern University, Chicago, IL 60611 USA; 3 DND CAT, Northwestern Synchrotron Research Center at the Advanced Photon Source, Argonne National Laboratory, 9700 South Cass Avenue, Argonne, IL 60439 USA; 4 X-ray Science Division, Advanced Photon Source, Argonne National Laboratory, 9700 South Cass Avenue, Argonne, IL 60439 USA; 5 Faculty of Biology, University of Belgrade, Belgrade, 11000 Serbia; 6 Serbian Academy of Sciences and Arts, Belgrade, 11000 Serbia

**Keywords:** Neuroprotection, CIPN, Amifostine, Cisplatin, XFM

## Abstract

Chemotherapy-induced peripheral neuropathy (CIPN) is a side effect of platinum-based chemotherapy and decreases the quality of life of cancer patients. We compared neuroprotective properties of several agents using an in vitro model of terminally differentiated human cells NT2-N derived from cell line NT2/D1. Sodium azide and an active metabolite of amifostine (WR1065) increase cell viability in simultaneous treatment with cisplatin. In addition, WR1065 protects the non-dividing neurons by decreasing cisplatin caused oxidative stress and apoptosis. Accumulation of Pt in cisplatin-treated cells was heterogeneous, but the frequency and concentration of Pt in cells were lowered in the presence of WR1065 as shown by X-ray fluorescence microscopy (XFM). Transition metals accumulation accompanied Pt increase in cells; this effect was equally diminished in the presence of WR1065. To analyze possible chemical modulation of Pt-DNA bonds, we examined the platinum L_III_ near edge spectrum by X-ray absorption spectroscopy. The spectrum found in cisplatin-DNA samples is altered differently by the addition of either WR1065 or sodium azide. Importantly, a similar change in Pt edge spectra was noted in cells treated with cisplatin and WR1065. Therefore, amifostine should be reconsidered as a candidate for treatments that reduce or prevent CIPN.

**Electronic supplementary material:**

The online version of this article (10.1007/s10571-019-00667-7) contains supplementary material, which is available to authorized users.

## Introduction

Chemotherapy-induced peripheral neuropathy (CIPN) is one of the common side effects of platinum-based chemotherapeutic drugs and a frequent cause for discontinuation of therapeutic treatments, affecting both the efficacy of the cancer treatment and the quality of life of cancer patients (Boyette-Davis et al. 2015; Carozzi et al. 2015; Chiorazzi et al. 2015; Kanat et al. 2017; Kerckhove et al. 2017; Ma et al. 2018; Miltenburg and Boogerd 2014; Paice et al. 2017; Starobova and Vetter 2017). Cisplatin (and now other Pt drugs such as oxaliplatin and carboplatin) has been used as a chemotherapeutic drug for 40 years, and it is still one of the most widely used chemotherapy drugs for lung, ovarian, testicular, bladder, and head and neck cancers (Dilruba and Kalayda 2016). The antitumor activity of cisplatin is mediated by its direct interaction with DNA, resulting in cross-linking and production of adducts leading to death of rapidly dividing tumor cells (Dilruba and Kalayda 2016; Marques et al. 2015; Wang and Lippard 2005). However, while molecular effects of platinum-based chemotherapeutic drugs are much studied in cancer cells, the spectra of their possible effects in non-dividing cells are less studied and less clear. The post-mitotic sensory neurons of the dorsal root ganglia (DRG) are especially vulnerable to cisplatin toxicity (Areti et al. 2014; Carozzi et al. 2015; Nicolini et al. 2015). Neuropathy caused by platinum drugs is associated not only with the formation of DNA adducts in genomic and mitochondrial DNA (Chiorazzi et al. 2015; Kanat et al. 2017; Starobova and Vetter 2017) but also with production of reactive oxygen and nitrogen species with consequent mitochondrial and endoplasmic reticulum stress and changes in axonal transport, etc. (Avan et al. 2015; Boyette-Davis et al. 2015; Canta et al. 2015; Carozzi et al. 2015; Chiorazzi et al. 2015; Foufelle and Fromenty 2016; Kanat et al. 2017; Kerckhove et al. 2017; McDonald et al. 2005; Nicolini et al. 2015; Waseem et al. 2018). For that reason, many treatments against CIPN include different types of antioxidants (Areti et al. 2014; Carozzi et al. 2010; Ma et al. 2018; Wang et al. 2018).

The aim of our study was to compare and evaluate possible neuroprotective properties of various antioxidants in an in vitro model of differentiated neurons NT2-N derived from pluripotent human embryonal carcinoma cell line NT2/D1. These cells resemble early embryonic stem cells in morphology, antigen expression patterns, biochemistry, developmental potential, and gene regulation (Andrews 1984). These cells have the ability to differentiate along the neural lineage during retinoic acid treatment yielding both neuronal and glial cell populations (Coyle et al. 2011). Terminally differentiated NT2-N display characteristics of post-mitotic polarized cells that express neurofilaments, generate action potentials and calcium spikes, express, release, and respond to neurotransmitters and form functional synapses (Guillemain et al. 2000; Hartley et al. 1999; Pleasure et al. 1992).

This model system allowed us to evaluate the potential of several antioxidants to reduce cisplatin-induced production of reactive oxygen radicals and neurotoxicity. Study endpoints included cell viability, oxidative stress and apoptosis as well as neurite outgrowth. Two of the most efficient modulators of cisplatin toxicity were found to be sodium azide (NaN_3_) and the thiol WR1065 (C_5_H_14_N_2_S·2HCl). While toxicity of NaN_3_ prohibits its use in vivo, a deeper look into its mechanism of action may still provide further insights into specific aspects of cisplatin toxicity. The thiol WR1065 was more efficient in protection of differentiated neurons, and importantly, it is also a drug in clinical use. WR1065 is active metabolite of amifostine (generic name)—a FDA approved drug also marketed under brand name Ethyol®. Its ability to accumulate more rapidly in normal tissues than in tumors (Yuhas 1979) led to its use to reduce the side-effects of radio- and chemotherapy on normal tissue in cancer patients, while not affecting the therapeutic efficacy of anti-cancer treatments (Lorusso et al. 2003; Senzer 2002; Small 2003; Wasserman et al. 2005). It should be noted that amifostine was tested against CIPN in several clinical trials (Albers et al. 2014; Beijers et al. 2012; Piccolo and Kolesar 2014; Schloss et al. 2013) with varied success, as well as in translational animal models (Treskes and van der Vijgh 1993; Yalcin et al. 2003). However, it is important to note that in these trials use of amifostine was secondary to chemotherapy and therefore the mode of its application varied. In order to simplify our studies, complicated by the multitude of unknowns when the totality of the effects of cisplatin and WR1065 is considered, we chose for this work the cell line NT2/D1. Not only are they a well-documented model system of human neurons in vitro (Coyle et al. 2011) but they have even been used in phase I and II clinical trials in stroke patients (Hara et al. 2008). This is possible because NT2/D1 cell line differentiates into terminally differentiated neurons with complete loss of tumor characteristics (Andrews 1984; Kurie et al. 1993; Spinella et al. 1999).

This in vitro study shows that WR1065 has considerable efficacy in protecting non-dividing neurons from adverse effects of cisplatin, suggesting that amifostine should be reconsidered as a part of anti-CIPN treatments. The recent development of a nanoparticle formulation of WR1065 (Pamujula et al. 2008) provides a possible solution for further improving targeting of this drug and increasing its dose locally while reducing its undesirable systemic effects such as hypotension.

We have used synchrotron X-ray fluorescence microscopy to quantify Pt accumulation in cisplatin treated cells in the presence or absence of WR1065. We also used X-ray spectroscopy to investigate the platinum L_III_ near edge spectrum. We found that the shape of the Pt spectra changes when a mixture of cisplatin and DNA is scanned alone or after an addition of some of the chemicals used in this study. These spectral modulations were very specific for samples that involved WR1065, and they were similar to Pt spectra in more chemically defined samples that included thiol groups (Marques et al. 2015; Provost et al. 2009; Sooriyaarachchi et al. 2016). In X-ray spectroscopy, near edge spectral modulations reflect the changes in the chemical environment of Pt and suggest alterations of chemical bonds. Because both WR1065 and NaN_3_ changed Pt spectra, it is possible that these two chemicals may protect neurons not only by acting as ROS scavengers, but also as chemical modulators of cisplatin–DNA interactions.

## Materials and Methods

### Chemicals

All chemicals and cell treatment agents were obtained from Sigma-Aldrich (MO, USA) unless otherwise specified. A stock solution of cisplatin was prepared in phosphate buffer saline (PBS) in a concentration of 1 mg/ml; WR1065 (2-[(3-aminopropyl)amino]ethanethiol; (C_5_H_14_N_2_S·2HCl)) the active form of the drug amifostine (usually administered to patients as the pro-drug WR2721) stock solution was also in 1 × PBS in concentration of 100 mM; stock solution of sodium azide was 2 M and prepared in water; histidine was prepared as 100 mM stock solution in 1 × PBS. Enzymes with antioxidant properties were prepared as follows: catalase at 1 mg/mL (2000–5000 U/mg) in 50 mM potassium phosphate buffer, superoxide dismutase (SOD) in 1 × Hanks’ Balanced Salt solution (Thermo Fisher Scientific, USA) at 10 u/µL. Cytotoxicity of each lot of cisplatin was tested separately, in order to evaluate the exact IC_50_ concentration of cisplatin. IC_50_ concentrations for differentiated NT2-N cells varied between 40 and 60 µM between different experiments. DCFH-DA (2′,7′-dichlorofluororescin diacetate) was obtained from Sigma-Aldrich and dissolved (50 mM) in dimethyl sulfoxide (DMSO) prior to use. Mitotic inhibitors used in experiments were: 1 mmol/L cytosine arabinoside, 10 mmol/L uridine, and 10 mmol/L 5-fluoro-5-deoxyuridine (all from Sigma-Aldrich, MO, USA).

### Cell Culture

The human embryonic teratocarcinoma cell line NTERA-2 cl.D1 (also known as NT2/D1) (ATCC® CRL-1973™) was cultured in Dulbecco’s modified Eagle medium (DMEM) supplemented with 10% fetal bovine serum (FBS), a mixture of antibiotics and antimycotics and 1 mM sodium pyruvate. Cells were grown in a humidified atmosphere containing 5% CO_2_ at 37 °C. SK-N-SH cells (ATCC® HTB-11™) were grown in DMEM medium supplemented with 10% fetal bovine serum (FBS), mixture of antibiotics and antimycotics and 1 mM sodium pyruvate. Cells were regularly checked for mycoplasma contamination using PCR with primers forward: ACTCCTACGGGAGGCAGCAGTA and reverse: TGCACCATCTGTCACTCTGTTAACCTC.

Proliferating NT2/D1 cells were induced to differentiate in cell culture by addition of 10 µmol/L all-*trans* retinoic acid (RA) for 4 weeks as previously described (Popovic et al. 2014). Following RA induction, cells were trypsinized and plated at sixfold lower density. After 2 days, differentiated neuron-like cells were detached from the plate by tapping mechanically on the side of the tissue culture plate and re-plated on Geltrex ® (Thermo Fisher Scientific, MA, USA) coated dishes. Over the following 4 to 7 days, cells were cultured in the presence of mitotic inhibitors: 1 µmol/L cytosine arabinoside, 10 µmol/L uridine and 10 µmol/L 5-fluoro-5-deoxyuridine. Terminally differentiated neurons are referred to as NT2-N cells throughout this work. For SK-N-SH cells, the neuronal phenotype was induced by incubation in low serum (1% FBS) cell culture medium supplemented with 10 µM RA for 3 days, as previously described (Niewiarowska-Sendo et al. 2015). Differentiated SK-N-SH cells were maintained in 5% FBS cell culture medium in 5% CO_2_ at 37 °C.

### MTS Assay

Undifferentiated NT2/D1 and SK-N-SH cells were seeded at a density of 5 × 10^3^ cells per well. Differentiated NT2-N neurons were seeded at a density of 3 × 10^4^ cells/well in transparent 96-well plate coated with Geltrex (Thermo Fisher Scientific, MA, USA) and cultured for 5 days in the presence of mitotic inhibitors as described above. Differentiated SK-N-SH cells were seeded at the same density and treated the day after, without the treatment with mitotic inhibitors. On the day of the experiment, cells were exposed to cisplatin (for IC_50_ assessment) or cisplatin alone or in the presence of 5 mM WR1065, 10 mM NaN_3_, 10 mM Histidine, 400 U Catalase or 150 U of Superoxide dismutase (SOD) for antioxidant evaluation. Incubation lasted for 1 h in DMEM in 5% CO_2_ at 37 °C for 1 h. After the treatment, cells were washed with cell growth media and cultured for additional 48 h in complete cell growth medium. Cell viability was assessed by a colorimetric assay using the CellTiter 96® AQueous One Solution Cell Proliferation Assay (Promega, Madison, WI, USA). The readings were done by Tecan microplate reader at 490 nm wavelength and analyzed by Magellan software, or by BioTek microplate reader, Synergy 2, using software Gen5. The experiments were done in six replicates and repeated in at least 3 independent experiments.

### DCF Assay for Oxidative Stress

3 × 10^4^ NT2-N cells per well were plated in dark 96-well plate. Dichlorofluorescein (DCF) assay adapted for microplate reader was used, as developed by others (Girard-Lalancette et al. 2009; Wang and Joseph 1999). On the day of analysis, the cells were washed with 1 × PBS and then incubated with 50 µM DCFH-DA in PBS per well in 5% CO_2_ at 37 °C for 30 min and then washed again in PBS. After the wash, the fresh medium with cisplatin, or cisplatin and WR1065 or NaN_3_ was added and fluorescence was measured on the Tecan microplate reader immediately after treatment administration. Kinetic readings were measured with excitation at 485 nm and emission at 530 nm for 180 min with 5 min per cycle setting. The data were exported to Excel (Microsoft); the area under the curve (AUC) was calculated as AUC = [*R*_1_/2 + sum (*R*_2_:*R*_n−1_) + *R*_n_/2] × CT, where *R*_1_ is the fluorescence reading at the initiation of the reaction, *R*_n_ is the last measurement, and CT = cycle time in minutes. The AUC was obtained by subtracting the AUC of the blank from that of a sample, expressed as net AUC = AUC_sample_ – AUC_blank_. In the case of treatment with cisplatin alone, the blank wells contained cells treated with DCFH-DA and culture medium only. In the case of concomitant treatment with cisplatin and WR1065 or NaN_3_, the blank wells contained cells treated with DCFH-DA and culture medium supplemented with WR1065 or NaN_3_. Each control and treatment were done in six replicates and repeated in 3 independent experiments.

### Western Blot

Total cell extracts were obtained by suspending cells in lysis buffer containing 1% Triton X-100, 50 mM Tris–HCl, pH 7.5, 250 mM NaCl, 5 mM EDTA, and protease inhibitor cocktail (Roche Diagnostics GmbH, Germany). Proteins were quantified by Bradford protein assay (Bio-Rad, USA). Samples containing 30 µg of total cell extract were separated by SDS-PAGE on 15% resolving gels and electro-transferred to Immobilon-P Transfer Membrane (Millipore, USA). After blocking with 5% nonfat milk in TBST (20 mM Tris, pH 7.4, 150 mM NaCl, 0.05% Tween-20) at room temperature for 1 h, the membranes were incubated overnight at 4 °C with the following primary antibodies: rabbit anti-Cleaved Caspase-3 (Asp 175) (Cell Signaling, USA diluted 1:1000), rabbit anti-PARP (Cell Signaling, USA diluted 1:500). The membranes were likewise incubated for 1 h at room temperature with mouse anti-GAPDH (Abcam, UK; diluted 1:5000). Afterward, the membranes were washed in TBST and incubated for 1 h at room temperature with the following secondary antibodies: horseradish peroxidase-conjugated goat anti-mouse or goat anti-rabbit IgG (Active Motif, USA; diluted 1:10,000). Immunoreactive bands were detected by chemiluminescence (Immobilon substrate; Millipore, USA).

### Apoptosis Assay

NT2-N cells were exposed to the IC_50_ concentration of cisplatin alone or in combination with 0.5 mM WR1065 and 24 h after treatments the cells were washed with cold PBS, detached from the plate using trypsin and resuspended at 1 × 10^6^ cells/ml in 1 × Annexin binding buffer. Five microliters of Annexin V (Annexin V, Alexa Fluor® 488 conjugate, Invitrogen™) and 5 µl of propidium iodide (PI—Invitrogen, 1 mg/ml) were added. The cells were gently mixed, incubated for 15 min in the dark at RT and analyzed by CyFlow® Space Partec using the PartecFloMax® software (Partec GmbH, Münster, Germany).

### Immunocytochemistry

The effects of cisplatin and WR1065 on cell morphology were analyzed using imunocytochemistry. NT2-N cells were plated on Geltrex® coated cover slips and treated as previously described. 40 µM concentrations of cisplatin alone or combined with 5 mM WR1065 were used. Cells were cultured for 24 h after 1 h treatment with cisplatin alone or cisplatin and WR1065. Cells were fixed in 4% paraformaldehyde in PBS for 20 min at room temperature. Fixed cells were permeabilized in 0.1% Triton X-100 in PBS and blocked in 1% bovine serum albumin, 10% normal goat serum and 0.1% Triton X-100 in PBS for 1 h at room temperature. The primary antibody against microtubule associated protein 2 (MAP2, Abcam-ab11267), specific for mature neurons was diluted 500 fold in PBS containing 1% BSA, 0.1% Triton X-100 and samples were incubated overnight at 4 °C. Cover slips were washed 3 times for 10 min in 0.1% Triton X-100 in PBS and incubated with anti-mouse secondary antibody conjugated with Alexa Fluor® 488 (Thermo Fisher Scientific, USA), diluted 1500 fold in PBS containing 1% BSA, 0.1% Triton X-100. Nuclei were stained with 0.1 mg/mL diamino phenylindole (DAPI) and coverslips mounted with mounting medium (Thermo Fisher Scientific, USA). Samples were viewed at Northwestern University Cell Imaging Core Facility, under Nikon A1R microscope (Nikon Corporation, Japan) equipped with multispectral detectors for spectral un-mixing and images recorded and analyzed using Nikon ND2 software.

### Neurite Outgrowth Assay

The quantification of neurites was done using Neurite Outgrowth Assay Kit (Chemicon NS220, Merck, Darmstadt, Germany). The experiments were performed according to the manufacturer’s instructions. Upon differentiation with RA, NT2-N neurons were collected after mechanical agitation as described and seeded on the insert membrane surface. The underside of the each insert membrane was coated with Geltrex® prior to seeding of 10^5^ differentiated cells per insert. The cells were cultured on insert membranes for 5 days in the presence of mitotic inhibitors as described above. The samples included control, cells treated with cisplatin and cells exposed to cisplatin and 5 mM WR1065 at the same time. After 1 h of treatment the cells were washed and incubated in complete media supplemented with mitotic inhibitors for additional 48 h. The staining and colorimetric quantification of neurites were performed as recommended by the manufacturer. The experiment was repeated three times, with NT2-N cells obtained from three independent differentiations.

### X-Ray Fluorescence Microscopy

Differentiated NT2-N cells were plated on Geltrex® coated silica nitride (Si_3_N_4_) windows (Silson, UK), cultured in the presence of mitotic inhibitors and treated as previously described. 40 or 100 µM concentration of cisplatin on its own or combined with 5 mM WR1065 was used for sample treatments. After a 1-h treatment, cells were washed and incubated in complete media for one more hour. Finally, samples were briefly washed in three changes in buffer suitable for subsequent cell cryo-preservation and cryo-imaging by X-ray fluorescence microscopy (261 mM glucose, 9 mM acetic acid in 10 mM Tris buffer, pH 7.4). Samples were flash frozen in liquid ethane. With the cisplatin concentrations and incubation timepoints used, most of the cells are expected to be alive at the moment of freezing.

X-ray fluorescence microscopy of frozen NT2-N cells on Si_3_N_4_ windows was done in combination with use of the cryo-jet at the beamline 2ID-D at the Advanced Photon Source of Argonne National Laboratory. The incident X-ray energy was tuned to 12.5 keV using a beam splitting Si(111) monochromator. The beam was focused to a diameter of 300 nm using a Fresnel zone plate. A single element silicon drift energy dispersive detector (Vortex EX, SII Nanotechnology, Northridge, CA) positioned at 90° to the incident beam was used to collect the fluorescence signal from samples. Several hundreds of microns areas of the sample were scanned with a step size of 1 µm and a per-pixel dwell time of 100 ms. Per pixel elemental concentration was obtained by comparison with the thin-film standards NBS-1832 and NBS-1833 from the National Bureau of Standards (Gaithersburg, MD). The analysis was performed using MAPS software (Vogt 2003).

The same samples were imaged at the Bionanoprobe instrument as well (Chen et al. 2014). Monochromatic 11.6 keV hard X-rays were used to excite fluorescence in cryogenically fixed cell samples, this time focused to a spot size of ~ 85 nm using Fresnel zone plates. The fluorescence spectra at each step of the scan were collected with a four-element silicon drift detector (Vortex ME-4, SII Nanotechnology). Data were fitted and quantified by comparison to a standard reference material (RF8-200-S2453, AXO Dresden GmbH) using the MAPS program (Vogt 2003).

### X-Ray Spectroscopy

X-ray spectroscopy data were collected at the Pt L_III_-edge (11,564 eV) using the bending magnet beamline of the DuPont-Northwestern-Dow Collaborative Access Team (5-BM-D) at the Advanced Photon Source of Argonne National Laboratory. X-ray energy scans were performed using a Si(1,1,1) double-crystal monochromator. Samples were lyophilized and the powder wrapped in Ultralene™ membrane (SPEX, Metuchen, NJ). Samples were measured both by fluorescence and in transmission geometry; a Pt metal foil was used for energy verification. Each spectra acquisition lasted about 10–25 min with several repetitions run for each sample in order to reach a good signal to noise ratio. Data were normalized using the program Athena (Ravel and Newville 2005).

The concentration of Pt in “chemical mixtures” was 100 µM and much less in cell samples; either is less than the ideal Pt concentration (~ 1 mM) for the analysis by X-ray absorption spectroscopy (e.g., according to (Provost et al. 2009)). Therefore, all chemical mixtures or cell samples were collected in small volume of PBS, frozen at − 70 °C and concentrated by lyophilization at − 50 °C. Dry powder was confined in “sacks” made of Ultralene™ membrane.

### Statistical Analysis

Calculations of cisplatin IC_50_ values and calculations of statistical significance for cell viability assays were done using GraphPad Prism Software 6.0 (GraphPad Software, Inc.). Student’s *t* test was used to evaluate statistical significance in all graphs presented.

## Results

### WR1065 and Sodium Azide Increase Neuronal Viability In Vitro in Co-treatment with Cisplatin

**Fig. 1:**
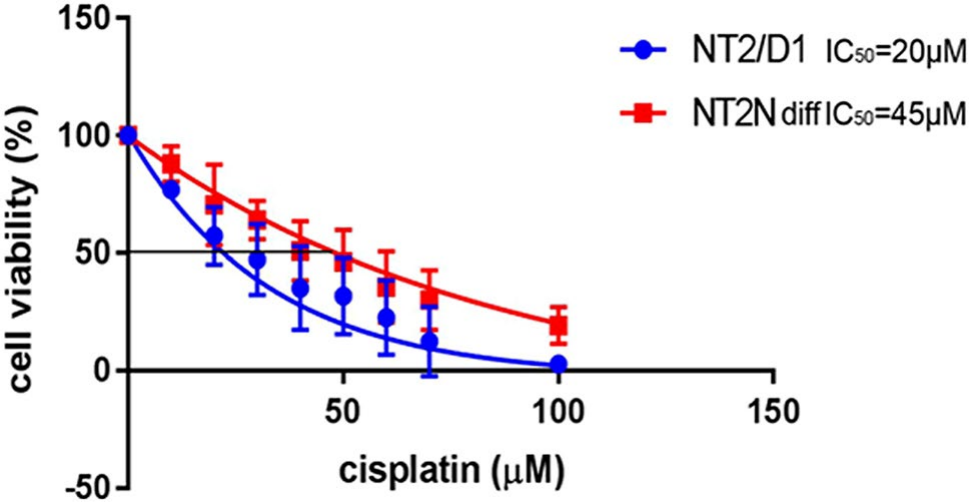
IC_50_ determination for undifferentiated (NT2/D1) and differentiated (NT2-N) cells. Undifferentiated and differentiated cells were exposed to increasing concentrations of cisplatin (from 10 to 100 µM) for 60 min and their viability was tested 48 h later by MTS assay

We evaluated cisplatin-induced cytotoxicity using non-differentiated NT2/D1 and differentiated NT2-N cells. Undifferentiated NT2/D1 cells, subclone derived from testicular teratocarcinoma, were two times more sensitive to cisplatin treatment, compared to differentiated NT2-N cells (Fig. 1). Similar results were obtained for another model system, SK-N-SH cell line derived from neuroblastoma, cells that also differentiate into neurons in the presence of RA (Niewiarowska-Sendo et al. 2015). Differentiated SK-N-SH cells displayed over a threefold increase in IC_50_ compared to undifferentiated cells (Supplemental Fig. 1). Cell differentiation especially in neuronal types of cells is often accompanied by decreased sensitivity to cisplatin (Lasorella et al. 1995). These data argue that human neurons obtained by in vitro differentiation of NT2/D1 cells are a good model system for analysis of mechanisms that underlie the process of neuronal damage induced by cisplatin and possible approaches to protect them from these effects. In all subsequent experiments, only the differentiated NT2-N cells were used.

Several different compounds were tested as possible modulators of cisplatin toxicity caused by oxidative stress in differentiated NT2-N cells. We tested the enzymes Superoxide dismutase (SOD) and catalase. SOD is known as an enzyme which converts superoxide (O_2_^•−^) into hydrogen peroxide (H_2_O_2_), while catalase removes H_2_O_2_ from cells (Anderson and Phillips 1999). Non-enzymatic scavengers of singlet oxygen—sodium azide and histidine (Hall and Chignell 1987; Lindig and Rodgers 1981; Zang et al. 1990) were tested as well. Finally, we have also tested the active thiol of amifostine—WR1065. Amifostine is a prodrug hydrolyzed by alkaline phosphatase (primarily in liver) into its active, cell permeable and cytoprotective metabolite WR1065. Amifostine was developed originally by the U.S. Army Anti-Radiation Drug Development Program at Walter Reed as a compound WR-2721 (PubChem CID2124; FDA UNII: ILA426L95O) and found to reduce genotoxic effects of radiation and chemotherapeutic drugs in vivo. WR1065 the active metabolite of amifostine is active in vitro and acts as a scavenger of reactive oxygen species that efficiently reduces the extent of DNA-damage (Bohuslavizki et al. 1999; Grdina and Sigdestad 1989; Nici et al. 1998).

**Fig. 2:**
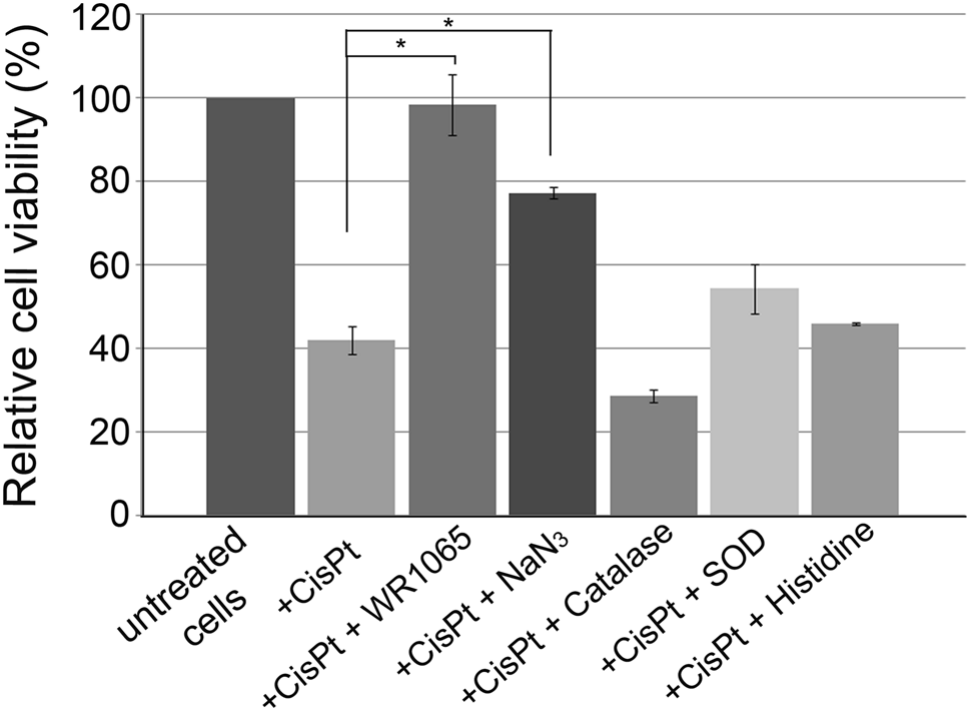
Relative viability of NT2-N cells upon treatment with cisplatin alone or in combination with different antioxidants. NT2-N cells were co-treated with IC_50_ concentration of cisplatin and 5 mM WR1065; 10 mM NaN_3_, 400 U catalase; 150 U SOD, or 10 mM histidine. Relative cell viability was calculated compared to untreated NT2-N cells which were set as 100%. Results are presented as the means ± S.E.M. of at least three independent experiments, each performed in six replicates. Mean values were compared with Student’s *t* test; *p ≤ 0.05

Cisplatin was used at IC_50_ concentration to treat differentiated NT2-N cells alone or in combination with the compounds listed: 5 mM WR1065, 10 mM sodium azide, 10 mM histidine, 400 U of catalase or 150 U of SOD. After 1 h, cells were washed and grown for an additional 48 h in complete media. A significant increase in cell viability was obtained by co-treatments with WR1065 and sodium azide. WR1065 at 5 mM concentration completely restored cell viability matching that of untreated cells. Sodium azide increased the viability of cisplatin-treated cells as well, reaching about 80% of that in untreated cells. Catalase, SOD and histidine had no statistically significant effect on reduction of cisplatin toxicity (Fig. 2).

### Evaluation of Cisplatin-Induced Oxidative Stress in the Presence of WR1065 and NaN_3_

**Fig. 3:**
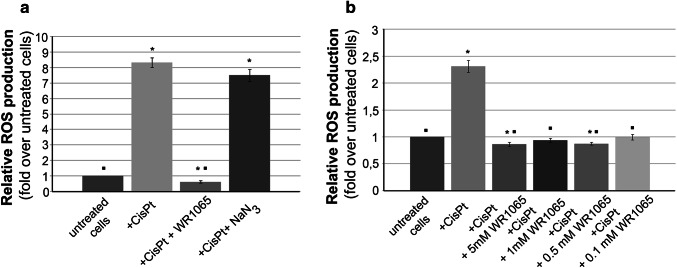
Effect of WR1065 and NaN_3_ on ROS production in NT2-N cells induced by cisplatin. The overall cisplatin-induced oxidative stress in NT2-N cells was quantified by intracellular DCF fluorescence. Relative ROS production was calculated compared to untreated NT2-N cells which were set as 1, and results are presented as fold over untreated cells. **a** NT2-N cells were co-treated with cisplatin and 5 mM WR1065 or 10 mM NaN3; **b** NT2-N cells were co-treated with cisplatin and different concentrations of WR1065. Results are presented as the means ± S.E.M. of at least three independent experiments. Mean values were compared using Student’s *t* test. Statistically significant differences (*p* ≤ 0.05) are indicated with an asterisk when samples were compared to control, or with a square when samples were compared to cisplatin exposed cells

In order to quantify production of reactive oxygen species caused by cisplatin treatments and explore how the reactive oxygen species (ROS) quantity correlates with cell viability, we conducted additional experiments (Fig. 3). For this purpose, we used dichlorofluorescein (DCF) assay using microplate reader (Wang and Joseph 1999). Briefly, the nonionic, nonpolar 2′,7′-dichlorofluororescin diacetate (DCFH-DA) freely crosses cell membranes of intact viable cells. Inside cells it is hydrolyzed enzymatically by esterases into non-fluorescent DCFH. However, in the presence of ROS, DCFH is oxidized to highly fluorescent dichlorofluoresscein (DCF). Therefore, the intracellular DCF fluorescence can be used to quantify the overall oxidative stress in cells. DCF assay for cells exposed to IC_50_ concentration of cisplatin demonstrated a significant production of ROS. This effect of cisplatin could be completely abolished by a co-treatment with 5 mM WR1065 (Fig. 3a). These data were not surprising considering the well-known antioxidant and DNA and protein protective activities of WR1065 in other cell types (Nagy et al. 1986; Treskes et al. 1992). Importantly, even when applied at a 50 times lower concentration (100 µM), WR1065 completely prevented cisplatin-induced ROS production in differentiated neurons (Fig. 3b). It should be mentioned that the peak plasma concentrations of WR1065 after administration of amifostine in patients are in a similar range and reach about 80 µM concentration and persist over a period of 7 to 10 h (Korst et al. 1997).

Interestingly, the co-treatment with cisplatin and 10 mM NaN_3_ did not significantly reduce ROS accumulation compared to cells treated with cisplatin alone (Fig. 3). These results suggest that the mechanism of cisplatin protection by 10 mM NaN_3_ is not based on ROS scavenging capacity of that molecule. NaN_3_ is a scavenger of singlet oxygen (Hall and Chignell 1987; Lindig and Rodgers 1981; Zang et al. 1990) but it is possible that singlet oxygen is not generated during cisplatin treatment. It will be particularly interesting to evaluate the mechanisms of NaN_3_ protection against cisplatin, knowing that modulation of ROS production plays no part in this activity. At the same time, it should be appreciated that the toxicity of NaN_3_ prohibits its use in vivo (Chang and Lamm 2003; Conger and Carabia 1977; De Flora 1979; Goshima et al. 2010; Hamza et al. 2017).

### WR1065 Prevents Apoptosis of Differentiated Neurons in Co-treatment with Cisplatin

**Fig. 4:**
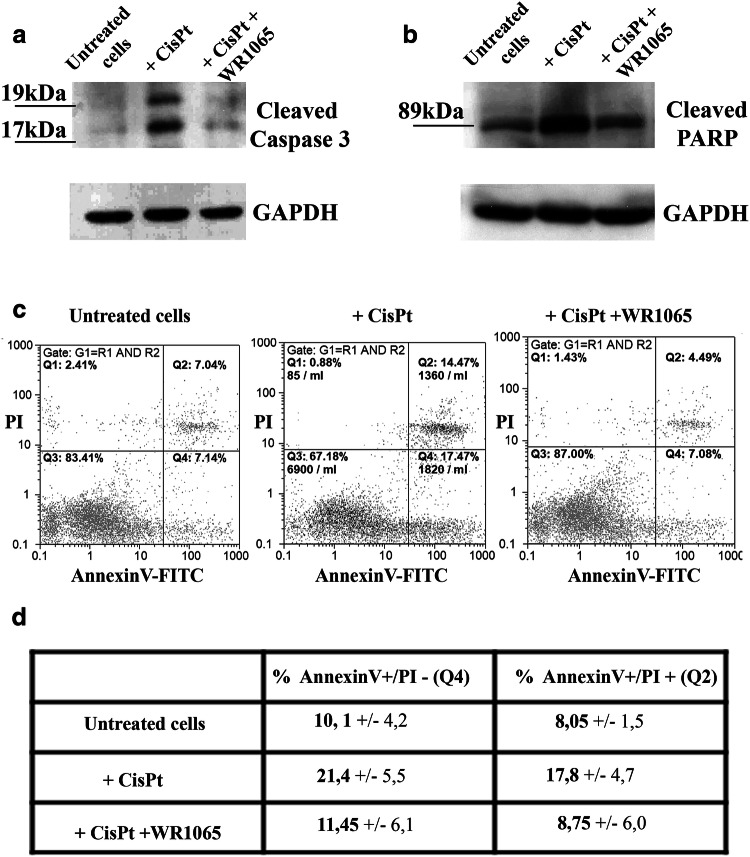
Analysis of apoptosis in NT2-N cells. Western blots (**a, b**) and flow cytometry analysis of apoptosis (**c, d**). For Western blots untreated cells and cells exposed to cisplatin alone (+ CisPt) or in combination with WR1065 (+ CisPt + WR1065) were harvested and their total protein content isolated. **a** Analysis of caspase 3 cleavage; activated Caspase 3 fragments are detected as 19 kDa and 17 kDa molecular weight bands. **b** Analysis of cleaved PARP; carboxy-terminal catalytic domain of PARP released upon cleavage generated 89 kDa band. GAPDH was used as a control of equal protein loading. **c** NT2-N cells were exposed to cisplatin alone or in combination with WR1065 and analyzed by flow cytometry after 24 h. Whole cells selected by gating were stained by PI and Annexin V and separated into quadrants. Q1: PI+/Annexin-cells are necrotic; Q2: PI+/Annexin + cells are late apoptotic cells; Q3: PI-/Annexin-cells are alive; Q4: PI-/Annexin V + cells are in early apoptosis. **d** Two independent flow cytometry experiments were done with similar outcomes—average values and standard deviation are presented in the table for early and late apoptotic cells

Apoptotic death of neurons induced by cisplatin is associated with changes in cellular behavior, including activation of caspases (Boyette-Davis et al. 2015). In order to test whether WR1065 has the ability to protect differentiated neurons from apoptosis caused by cisplatin, we evaluated the level of cleaved caspase 3 in NT2-N cells exposed to cisplatin alone or in combination with WR1065. Caspase-3 is one of the key executioners of apoptosis, as it is either partially or completely responsible for the proteolytic cleavage of many of the key cellular proteins, including the nuclear enzyme poly ADP ribose polymerase (PARP). Activation of caspase-3 requires proteolytic processing of its inactive zymogen into activated p17 and p12 fragments detected as 19 and 17 kDa molecular weight bands. Accordingly, Western blots were done for detection of caspase-3 and its activated fragments and for its main cleavage target (PARP) as indicators of apoptosis. Activated caspase-3 was increased in NT2-N upon treatment with cisplatin (lane + CisPt, Fig. 4a). This effect was less appreciable in the presence of 5 mM WR1065. In particular, when NT2-N cells were treated simultaneously with cisplatin and WR1065 the level of cleaved caspase 3 was similar to that observed in untreated cells (compare lanes untreated cells and + CisPt + WR1065, Fig. 4a). Similarly, exposure to cisplatin alone has led to an increased level of cleaved-PARP in NT2-N cells (lane + CisPt, Fig. 4b), while co-treatment with cisplatin and WR1065 reduced the level of cleaved PARP to that in control cells (lanes untreated cells and + CisPt + WR1065, Fig. 4b). Thus, WR1065 protects differentiated neurons from initiation of apoptosis by cisplatin in vitro.

Finally, flow cytometry was done with differentiated NT2-N cells treated with cisplatin alone or in combination with WR1065. Annexin V/propidium iodide double-staining analysis revealed that WR1065 prevented induction of apoptosis (Fig. 4c). The number of both early apopototic Annexin V+/PI− cells and late apoptotic Annexin V+/PI + cells was increased 24 h after treatment with cisplatin. At the same time, cells exposed to cisplatin and WR1065 together showed reduced levels of cells in both early and late apoptosis (Fig. 4c, d) compared to cisplatin treatment alone.

### Analysis of Neuronal Morphology and Neurite Outgrowth Assay

**Fig. 5:**
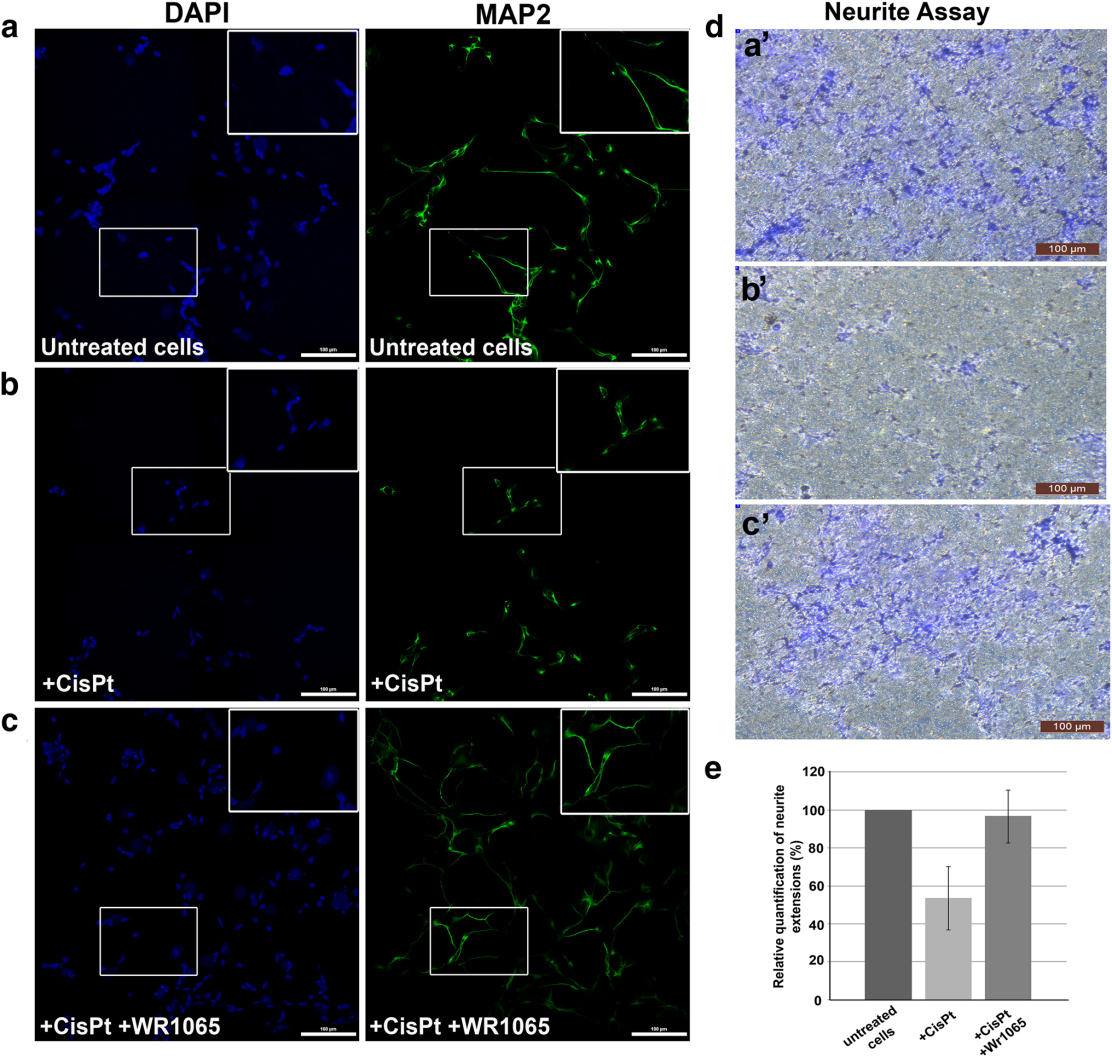
NT2-N morphology and neurite outgrowth assay. Panels **a** to **c** show MAP2 immunocytochemistry of differentiated NT2-N cells – untreated control cells (**a**), cells treated with 40 µM cisplatin alone (**b**), cells co-treated with 40 µM cisplatin and 5 mM WR1065 (**c**). Cell nuclei were stained with DAPI (represented in blue) while MAP2 staining is shown in green. Boxed regions in **a**, **b**, and **c** are enlarged in upper right corner of each panel. Scale bars represent 100 microns. Neurite Outgrowth Assay (**d, e**): 10^5^ NT2-N cells were seeded on each insert for Neurite Outgrowth Assay and allowed to extend neurites for 48 h. Then, cells were stained with Neurite Stain Solution for 30 min and the cell bodies were removed from the upper part of the insert. Neurite extensions were visualized using inverted microscope focused on the underside of the insert’s membrane (**d**: **a’**—untreated cells; **b’**—cells treated with 40 µM cisplatin; **c’**—cells treated with 40 µM cisplatin + 5 mM WR1065). Magnification 10×, scale bar 100 µm. **e** shows relative quantification of neurite extensions by spectrophotometry at 562 nm, according to manufacturer’s instructions. Relative quantification was calculated with untreated NT2-N cells value set at 100%. Results are presented as the means ± S.E.M. of three independent experiments with cells obtained from three independent NT2 cell differentiation experiments

The morphology of NT2-N cells in the presence of cisplatin alone and in co-treatment with WR1065 was evaluated by microscopy; identity of cells as differentiated neurons was confirmed by surface marker expression. Immunocytochemistry was done using the neuronal marker—microtubule associated protein-2 (MAP2) that belongs to the microtubule-associated protein family. The proteins of this family are involved in microtubule assembly, which is an essential step in neuritogenesis (Sanchez Martin et al. 2000). We found that neuronal morphology is compromised in the presence of cisplatin, where the length of neurites in treated cells is shorter compared to untreated cells (Fig. 5, compare panels a and b). On the other hand, the neurons treated with IC_50_ concentration of cisplatin and WR1065 resembled the morphology of untreated cells (Fig. 5, compare panels b and c).

The neurite outgrowth assay used in this study allows characterization of neurite formation, composition and behavior in response to treatments with different chemical agents. Cells are seeded on a permeable membrane that allows for discrimination between neurites and cell bodies, as projecting neurites pass easily through the membrane pores, while cell bodies do not. The staining of neurites with the neurite stain solution showed that the treatment with cisplatin notably affected the neurite outgrowth (Fig. 5, compare panels d-a’ and d-b’); again, co-treatment with WR1065 decreased cisplatin-induced damage (Fig. 5, compare panels d-b’ and d-c’). The colorimetric quantification of the neurite development was performed by reading the absorbance on 562 nm (Fig. 5e).

### X-Ray Fluorescence Microscopy (XFM)

Numerous studies with cycling cells in cell culture and cisplatin were done in the past; the toxic effects of cisplatin on cells were associated with the formation of platinum-DNA adducts as well as damage to other cellular components and organelles—from mitochondria to axonal transport (Avan et al. 2015; Boyette-Davis et al. 2015; Canta et al. 2015; Carozzi et al. 2015; Chiorazzi et al. 2015; Foufelle and Fromenty 2016; Kanat et al. 2017; Kerckhove et al. 2017; McDonald et al. 2005; Nicolini et al. 2015; Starobova and Vetter 2017; Waseem et al. 2018). In order to investigate the subcellular location of platinum in cisplatin-exposed NT2-N cells, we chose to use X-ray fluorescence microscopy (XFM)—the only imaging technique that can be used for direct visualization and quantification od chemical elements, native to cells or exogenously introduced into them (Bourassa et al. 2014; Kim et al. 2010; Paunesku et al. 2006, 2012; Wang et al. 2014; Weekley et al. 2013; Wolford et al. 2010). Moreover, XFM has been used previously to image Pt distribution in spheroids (Zhang et al. 2012) and cycling cells (Hall et al. 2003), and to uncover the idea that passive permeation of platinum-based drugs across the plasma membrane makes up a significant portion of their cellular uptake (Eljack et al. 2014).

**Fig. 6:**
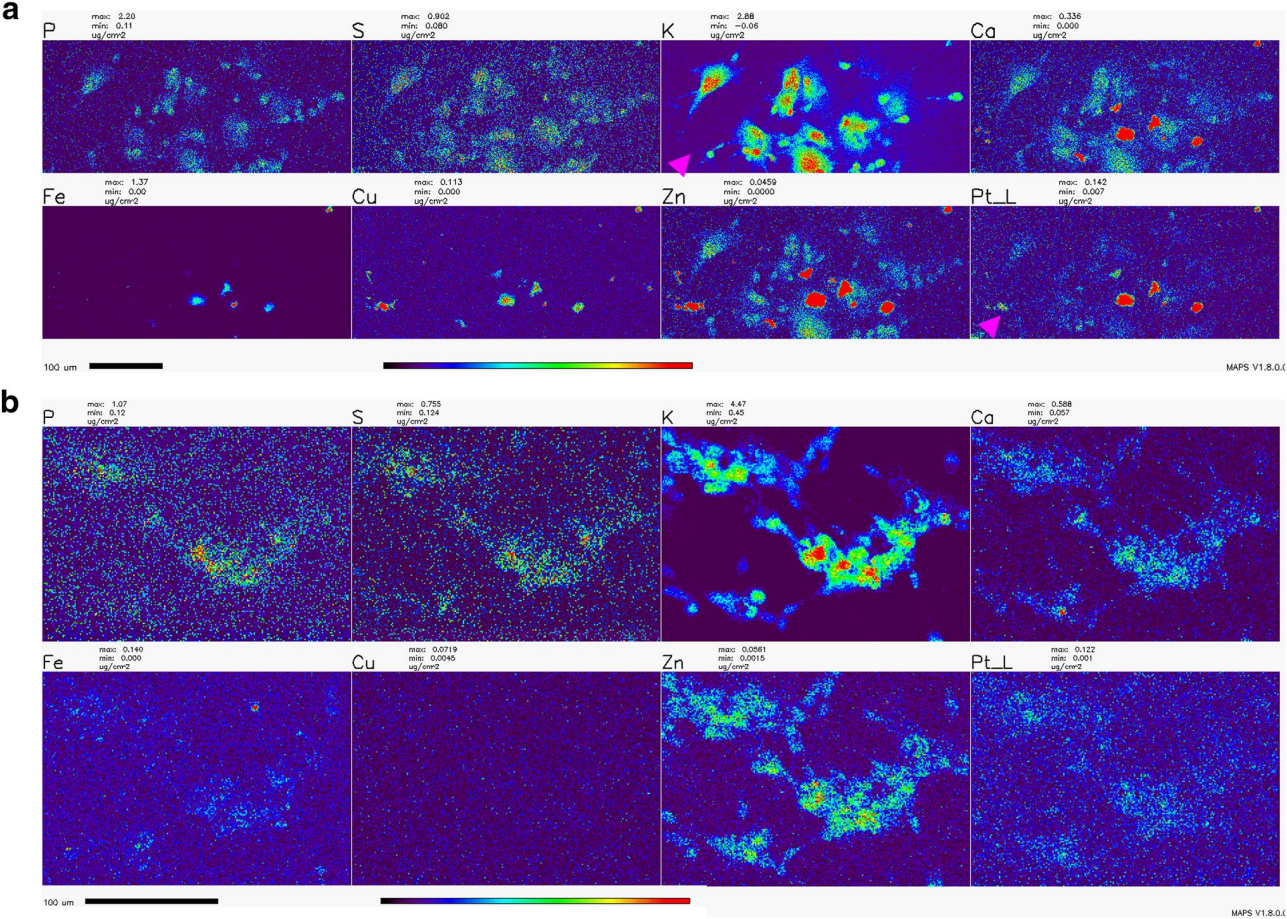
Overview XFM scans of NT2-N cells treated with cisplatin with and without the addition of WR1065. **a**—cisplatin only; **b**—cisplatin and WR1065 co-treatment. Note the heterogeneity between cells and that cells with low K signal intensities have highest Pt pixel intensities (magenta arrowheads). The same group of cells also had high Ca, Fe, Cu and Zn signals. In addition, cells with an apparently high Pt signal are relatively abundant in cisplatin alone treatment (**a**), compared to cisplatin-WR1065 combined treatment (**b**). Scale bars are 100 microns, color bar indicates that per pixel concentrations for each element range from black (no signal) to red (highest signal for a given element). Concentration ranges per-pixel are indicated above each element’s map, e.g., Pt per-pixel concentrations based in top panel (cisplatin alone) range from 0.007 µg/cm^2^ (dark blue pixels) to ≥ 0.142 µg/cm^2^ (red pixels); corresponding span of Pt per-pixel signal intensities in cisplatin-WR1065 panel (bottom) is 0.001 (dark blue pixels) to ≥ 0.122 µg/cm^2^ (for the few red pixels in this image). For mean per pixel elemental concentrations see Table 1

**Fig. 7:**
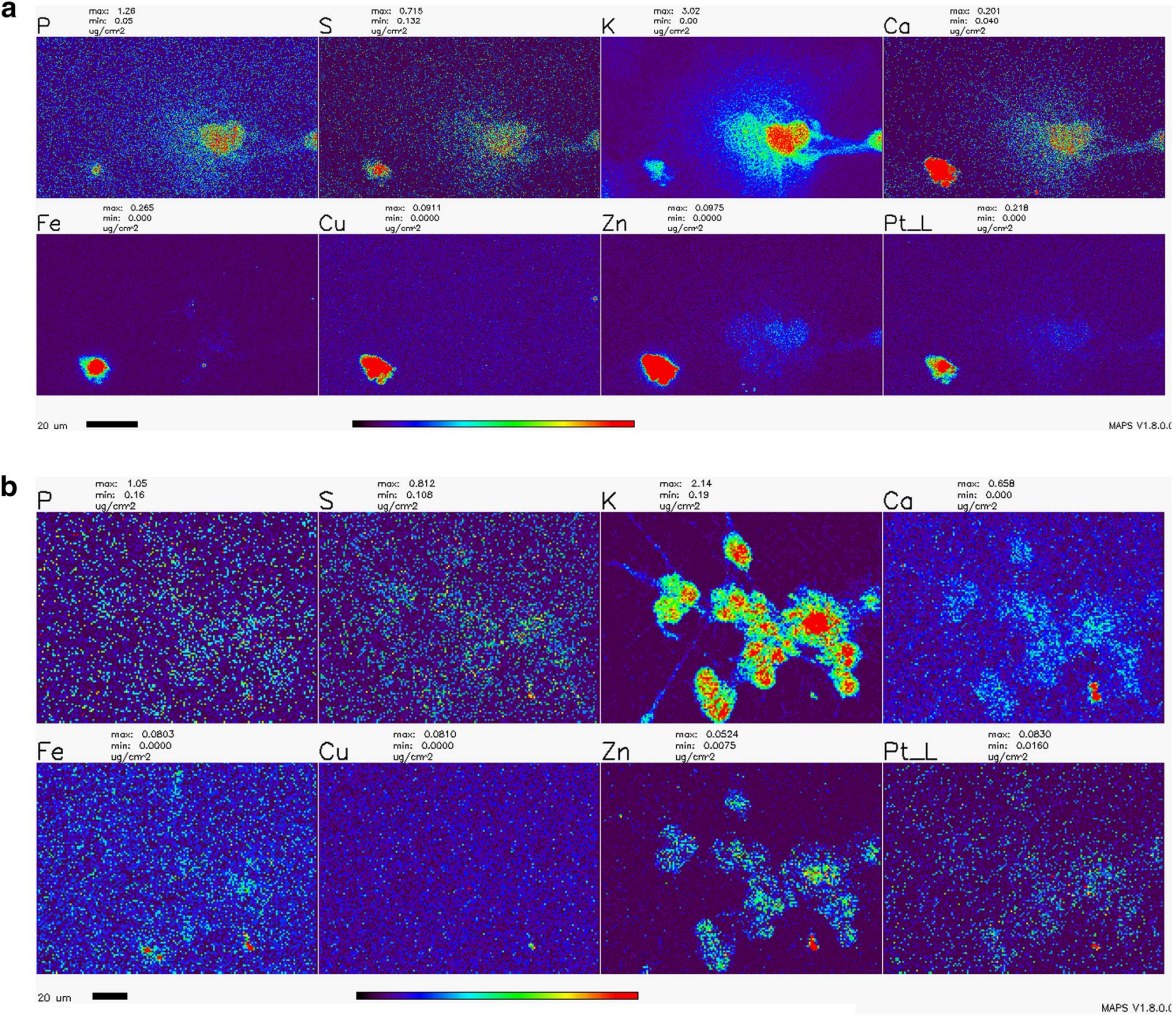
Detailed XFM scans of NT2-N cells treated with cisplatin with and without addition of WR1065. **a**—cisplatin only; **b**—cisplatin and WR1065 co-treatment. Note the heterogeneity between cells and that cells with low K signal intensities the have highest Pt pixel intensities; the same cells have high Ca, Fe, Cu and Zn signals (most evident in cisplatin only—**a**). Scale bars are 20 microns, the color bar indicates that elemental concentrations for each element range from black pixels (no signal) to red pixels (highest signal for a given element). Concentration ranges are indicated above each element’s map, e.g., Pt concentration in **a** (cisplatin treatment) ranges from 0.000 µg/cm^2^ (black pixels) to ≥ 0.218 µg/cm^2^ (red pixels); in cisplatin-WR1065 treatment example (**b**), Pt signal goes from 0.016 µg/cm^2^ (dark blue pixels) to ≥ 0.063 µg/cm^2^ (red pixels). For mean elemental concentrations see Table 1. For a high detail scan of few cisplatin treated cells see Supplemental Fig. 3

The distribution of Pt in non-dividing cells was not evaluated in the past by XFM, and the effects of WR1065 on Pt distribution in cells were never explored by XFM in any cell type. In this work, we imaged the Pt distribution using XFM in cryogenically prepared cell samples using the 2IDD beamline run with a cryo-jet and the Bionanoprobe instrument run under cryogenic conditions. This approach to XFM permitted us to image potassium—a highly diffusible ion that is retained in high concentrations only in viable cells (LeFurgey et al. 1990), and in that way document the viability for each one of the cells in the field of view. The concentration of cisplatin used to prepare samples for XFM imaging was 100 µM in order to allow for detection of Pt in cells. At the timepoints used to prepare samples, cell death was infrequent, especially in samples co-treated with cisplatin and WR1065. Importantly, we noted a clear inverse correlation between cells that accumulated Pt, mostly in nuclei, and cells that retained physiologic potassium concentration (Figs. 6, 7; Table 1). We did not notice an increased concentration of sulfur in samples treated with 5 mM WR1065 despite its sulfur content (Figs. 6, 7; Table 1).

**Table 1:** Elemental concentrations of Pt, trace elements and main biological elements in healthy and dying cells exposed to cisplatin or cisplatin and WR1065

Element—mean µg/cm^2^ elemental concentration per pixel	P	S	K	Ca	Fe	Cu	Zn	Pt
NT2-N cells treated with cisplatin alone								
K low, Pt high cells	0.150	0.270	0.524	0.532	1.290	0.132	0.848	0.153
	0.117	0.157	0.435	0.508	0.337	0.061	1.331	0.239
K high, Pt low cells	0.677	0.295	2.209	0.106	0.004	0.001	0.012	0.014
	0.569	0.259	1.088	0.062	0.002	0.000	0.011	0.011
NT2-N cells treated with cisplatin and WR1065								
K low, Pt high cells	0.797	0.434	0.891	10.387	0.155	0.034	0.377	0.069
	0.150	0.154	0.896	0.088	0.004	0.000	0.010	0.025
K high, Pt low cells	0.151	0.136	1.586	0.082	0.002	0.000	0.011	0.006
	0.193	0.160	1.676	0.100	0.006	0.001	0.013	0.008

Two examples each are provided for cells with Pt accumulation in cell nuclei and low K concentration and cells which did not accumulate Pt and retained high concentration of K for each treatment condition: 100 µM cisplatin alone or in combination with WR1065. In the latter case, neither the maximal Pt concentration nor the minimal K concentration were as pronounced as in cells treated with cisplatin alone. These data were extracted from regions of interest analyses of XFM scans presented in Figs. 6 and 7; they were also corroborated by higher-resolution scans in Supplemental Fig. 3

XFM imaging was conducted at a 300 nm resolution at beamline 2IDD, using a cryo-jet and an incident X-ray energy of 12.5 keV. Outlines of clustered viable NT2-N are clearly visible because of their potassium content. This image also reminds us that in differentiated NT2-N cells, in addition to neuronal type cells, one also finds “giant epithelial-like” cells. These cells contribute to data in the ROS readouts and MTS assay as well and only in this type of imaging one can both differentiate between the two cell sub-populations and quantify Pt in cells at the same time. Large area overview scans of NT2-N neurons treated with cisplatin alone or in combination with WR1065 were done, followed by more detailed scans of a few cells at a time (Figs. 6, 7); moreover, even higher-resolution scans of individual cells were done using the Bionanoprobe instrument (Supplemental Fig. 3). The beam spot size at the Bionanoprobe beamline is about 80 nm, as explained in the Methods section. NT2-N cells were treated with 100 µM cisplatin for 1 h, washed and incubated for additional 2 h, or treated with 100 µM cisplatin and 5 mM WR1065 for 1 h before they were washed and incubated for additional 2 h. Accumulation of Pt in cells was heterogeneous and always inversely proportional to the concentration of potassium. In large overview scans (Fig. 6), one can appreciate that relatively few cells accumulated Pt (7 of 36 cells for cisplatin only treatment), especially in co-treatment with WR1065 (2 of 57 for cisplatin and WR1065 treatment); more detailed scans (Fig. 7; Table 1) allowed us to see that in the presence of WR1065, presence of Pt inside cells was lower in both K high and K low cells. Another notable finding is that in dying cells with low K/high Pt signal concentrations of Ca, Fe, Cu and Zn are also elevated (Figs. 6, 7; Table 1). This effect of cisplatin was also less pronounced in the presence of WR1065. Interestingly, in prostate cancer cell lines, zinc supplementation was found to increase cancer resilience to chemotherapy (Kratochvilova et al. 2017).

Because we have noticed redistribution of transition metals including Ca in cells with low K and high Pt signal, and because Ca is important in maintenance of cellular homeostasis, we also explored whether apoptosis in Pt treated cells would be altered in the presence of Ca chelator BAPTA-AM. Two independent flow cytometry experiments (Supplemental Fig. 4A, B), however, did not support this idea—the presence of BAPTA-AM additionally increased apoptosis of NT2-N cells. Finally, we also imaged calcium itself in SK-N-SH cells using the ratiometric calcium dye FURA2 (Supplemental Fig. 5). In cells exposed to cisplatin, but not in controls or cells treated with cisplatin and WR1065, we noted a higher accumulation of Ca in some of the cells. At the same time, cells that appeared in final stages of apoptosis showed decreased calcium content. It is possible that a temporary increase in Ca concentration is noted only in cells that are already destined for apoptosis, and because of that attempts to regulate Ca homeostasis alone cannot rescue cells from cisplatin-induced programmed cell death.

### Analysis of Chemical Changes of Pt Using X-Ray Absorption Spectroscopy

**Fig. 8:**
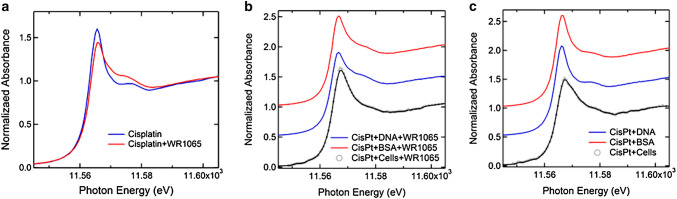
X-ray spectroscopy of Pt L_III_ edge in samples prepared by mixing cisplatin alone or with WR1065 with complex biological molecules DNA and BSA or by treating whole NT2-N cells with cisplatin with or without WR1065. **a** Comparison of Pt edge in cisplatin alone with Pt edge in a mixture of cisplatin and WR1065. Energy shift ΔE = 0.35 ± 0.04 eV suggests a change in the chemical environment of Pt when cisplatin is mixed with WR1065. **b** Comparison of Pt edge in cisplatin and WR1065 treated cells vs. mixtures of cisplatin and WR1065 and salmon sperm DNA (ΔE = 0.70 eV) or BSA (ΔE = 0.40 eV). **c** Comparison of the Pt edge in cisplatin treated cells vs. mixtures of cisplatin and salmon sperm DNA (ΔE = 0.60 eV) or BSA (ΔE = 0.45 eV)

X-ray absorption spectroscopy (XAS) can be used to investigate chemical changes in Pt in biological samples, and the literature has several examples of studies where gradual reduction of Pt(IV) into Pt(II) inside cells was followed (e.g., Hall et al. 2003). More frequently, Pt spectroscopy is done on chemically simpler samples such as mixtures of cisplatin and DNA oligonucleotides or even mono or di-nucleotides, alone or in combination with additional small molecules (Marques et al. 2015; Provost et al. 2009; Sooriyaarachchi et al. 2016). We have explored Pt in our samples using XAS at the DND-CAT beamline 5BMD at the APS. For each sample, 6–12 measurements were averaged to produce the graphs shown in Fig. 8. A complete list of samples and spectroscopy setups are provided in Supplemental Table 1.

Cell-free samples were prepared by mixing the cisplatin solution with purified salmon sperm DNA; this mixture was divided into aliquots and to some of them we added NaN_3_ or WR1065. This comparison of spectra (Supplemental Fig. 6) shows that changes in the Pt edge after addition of NaN_3_ differ from those connected with the addition of WR1065. This is not surprising considering that the latter probably arise from platinum sulfur chemical bonds (e.g., compare the shape of the cisplatin-WR1065 spectra (Fig. 8a, Supplemental Fig. 6) with those for platinum and thiosulfate (Sooriyaarachchi et al. 2016) or glutathione (Marques et al. 2015)). NT2-N cells treated with cisplatin with or without addition of WR1065 were freeze-dried and lyophilized to obtain powdered cell samples for spectroscopy. Differentiated cells were exposed to cisplatin (160 µM) for 1 h, with or without addition of 5 mM WR1065. Pt edge spectra of cell samples (Fig. 8) were compared with measurements taken on samples prepared by combining salmon sperm DNA and cisplatin or BSA with cisplatin alone or in combination with WR1065. All of these spectra show a specific change in shape of the Pt edge in samples co-treated with WR1065, regardless of the type of biological molecule present in the mixture, or presence of whole cells. Broadening of the Pt L_III_ edge when cisplatin treated cells are compared with mixtures of cisplatin and DNA or protein alone suggests increase in chemical disorder; Pt edge energy differences for cells to DNA or BSA comparisons are similar in the presence (Fig. 8b) or absence (Fig. 8c) of WR1065. Addition of WR1065 sharply alters the shape of the Pt edge in spectra of DNA-cisplatin and BSA-cisplatin samples. In cell samples, the presence of WR1065 in cisplatin treated cell samples led to a slightly more ordered edge structure (Fig. 8b). While one could consider that heterogeneity in Pt accumulation in cells in cisplatin treated sample could be responsible for a less orderly Pt XANES in the absence of WR1065, same change in Pt edge was obtained when whole cell homogenates were mixed with cisplatin alone or with WR1065 (data not shown). Moreover, the shape of the Pt edge change upon addition of WR1065 is similar to that observed in the literature for cisplatin mixed with glutathione (GSH) presumably due to interactions between Pt and sulfur (Marques et al. 2015; Provost et al. 2009; Sooriyaarachchi et al. 2016). Thiol groups of amifostine/WR1065 and glutathione (Marques et al. 2015) are the only sources of sulfur in these two compounds (for molecular structures see Supplemental Fig. 2).

## Discussion

The effects of cisplatin on living cells are generally considered to be associated with genomic and mitochondrial DNA damage and the creation of DNA adducts (Chiorazzi et al. 2015; Johnstone et al. 2016; Kanat et al. 2017; Starobova and Vetter 2017; Wang and Lippard 2005) but its side-effects are often associated with oxidative stress (Mercantepe et al. 2018; Wang et al. 2018). Oxidative stress, nitro-oxidative stress and mitochondrial impairment are often noted as possible factors in the etiology of cisplatin-induced neuronal injury (Areti et al. 2014; Carozzi et al. 2010; Ma et al. 2018; Maj et al. 2017; Waseem et al. 2018). Many different agents have been tested as possible interventions (Albers et al. 2014; Avan et al. 2015; Freyer et al. 2017; Fu et al. 2017; Glimelius et al. 2018; Kerckhove et al. 2017; Mishra and Alsbeih 2017; Piccolo and Kolesar 2014; Schloss et al. 2013), but a clear view of their mechanism of action is still lacking. In this work, several small molecules (histidine, sodium azide and WR1065) and proteins (SOD and catalase) were tested as possible modulators of cisplatin effects on differentiated, non-dividing neurons in cell culture. Sodium azide and WR1065—the active thiol form of amifostine, were found to be the most effective among them with respect to preservation of cell viability (Sodium azide and WR1065, Fig. 2) and ROS accumulation (WR1065, Fig. 3). Moreover, changes in cell morphology and neurite outgrowth in the presence of WR1065 were explored (Fig. 5), while several different X-ray based techniques (XFM and X-ray spectroscopy) were used as well in order to explore cellular heterogeneity in response to cisplatin and WR1065 treatment.

Sodium azide is often used as non-enzymatic scavenger of singlet oxygen (Hall and Chignell 1987; Lindig and Rodgers 1981; Zang et al. 1990). However, this is a reactive molecule that can be both oxidized or reduced and produce N_2_, NO, NO_2_, and N_2_O or N_2_H_4_ and NH_3_ (Dalmia et al. 1995). In a biological milieu, sodium azide is known as a poison (Chang and Lamm 2003), a mutagen (Conger and Carabia 1977; De Flora 1979), a possible source of oxidative stress (Hamza et al. 2017) and a modulator of axonal transport, similar to cisplatin itself (Goshima et al. 2010). Interestingly, in this study, sodium azide reduced cytotoxicity of cisplatin in vitro, as concluded by MTS assay. While its significant toxicity precludes its use in vivo, molecular and biochemical studies involving sodium azide and cisplatin may provide new insights into the exact nature of cisplatin toxicity and possible new ways to combat it.

The thiol WR1065, a metabolic product of amifostine, is regarded as a scavenger of free radicals, especially those generated in tissues exposed to chemotherapeutic agents or radiation (Giannopoulou and Papadimitriou 2003). However, its cytoprotective capacity is often associated with other mechanisms of action as well, for example, activation of mitochondrial superoxide dismutase (Murley et al. 2007, 2011), suppression of hyper-recombination (Dziegielewski et al. 2010), protection of lipophagy (Koukourakis et al. 2018), stimulation of polyamine synthesis (Mitchell et al. 1998), etc. Amifostine, a prodrug that is metabolized into WR1065, was tested in clinical studies as a protector against CIPN (Albers et al. 2014; Beijers et al. 2012; Piccolo and Kolesar 2014) with variable success. We hope that this more in-depth exploration of interactions between differentiated neurons, cisplatin and WR1065 may help to unravel some of the potential reasons for successes and failures of clinical studies. New nanoparticle formulations of amifostine and WR1065 may provide better targeting approaches leading to accumulation of WR1065 where it can be most beneficial. In this study, WR1065 not only reduced ROS accumulation and neuronal cell death but it also protected neuronal morphology and neurite outgrowth in the presence of cisplatin.

We have used X-ray fluorescence microscopy to image Pt accumulation in differentiated neurons exposed to cisplatin alone or in combination with WR1065. XFM maps generated (Figs. 6, 7 and Supplemental Fig. 3) demonstrate high cell to cell heterogeneity and an inverse relationship between loss of K (associated with cell death) and accumulation of Pt in individual cell nuclei. In WR1065 co-treated samples fewer cells had Pt accumulation and K loss and both phenomena were less pronounced (Figs. 6, 7; Table 1). Interestingly, we have also found increased accumulation of Ca, Fe, Cu and Zn in dying cells—this was not noted previously because no studies in the past used XFM for cell imaging for such large numbers of cells as we did in this study (Fig. 6). Interestingly, the presence of WR1065 also decreased this Ca, Fe, Cu and Zn accumulation in Pt accumulating, dying cells (Fig. 6).

While WR1065 has clear effects on ROS in cisplatin-treated cells and preservation of cell viability, we cannot exclude the possibility that the protective action of WR1065 (C_5_H_14_N_2_S·2HCl) and sodium azide (NaN_3_) may also depend on their ability to modulate chemical interactions between cisplatin Pt(NH_3_)_2_Cl_2_ and DNA. Other thiol compounds such as sodium thiosulfate for example have been studied in combination with cisplatin by extended X-ray absorption fine structure (Sooriyaarachchi et al. 2016). Authors of that study used solutions of cisplatin alone or mixed with sodium thiosulfate (Na_2_S_2_O_3_) to discover the formation of the four-coordinate Pt(II) species [Pt(S_2_O_3_)_4_]_6_ they named the tetrakis complex; nevertheless, the authors question the possibility of its formation in vivo and inside cells. The presumed mode of action of thiosulfate in the in vivo situation suggests that it sequesters and inactivates hydrolysis products of cisplatin, leaving un-sequestered active platinum species with anti-cancer activity. At this moment, thiosulfate is tested as a protector against hearing loss in cisplatin treated children (Freyer et al. 2017). Interactions with other sulfur-containing compounds (including the amino acid cysteine) were studied by X-ray spectroscopy as well (Provost et al. 2009). In addition, still other have investigated sulfur–DNA–platinum interactions by X-ray spectroscopy before, imaging cisplatin in complexes with adenine and guanine nucleotides and dinucleotides on their own or in combination with GSH (Marques et al. 2015). It should be noted that the native concentration of glutathione in cells is about 5 mM (equivalent to the concentration of WR1065 used in these experiments). We have used X-ray spectroscopy to evaluate not cisplatin adducts of adenine and guanine cisplatin, but rather more “natural” complexes between cisplatin and long molecules of DNA—its primary intracellular target. We have compared Pt L_III_ near edge spectra for cisplatin and DNA mixtures on their own and after mixing them with sodium azide or WR1065 (Fig. 8, Supplemental Fig. 6). Modulation of the spectra was evident with both chemicals and distinctly different. While we do not attempt to discern which exact chemical bonds of Pt are involved in these complexes, participation of sulfur from WR1065 in these complexes seems to be suggested by the shape of the Pt edge spectrum (Fig. 8), resembling the literature for X-ray absorption spectroscopy for complexes of Pt and other sulfur compounds (Marques et al. 2015; Provost et al. 2009; Sooriyaarachchi et al. 2016).

In conclusion, we cannot yet assess the most important (or even primary) mode by which WR1065 protects against cisplatin: Is it prevention from oxidative damage, or prevention of uptake (or improvement of removal) of Pt from cells, or is the chemical modulation of Pt most important for WR1065’s protection against cisplatin caused apoptosis, or is it some combination of these? This work suggests that it is necessary to continue to follow all three paths in this exploration.

## Conclusions

Neuropathic side effects of cisplatin are associated with many different cellular pathways; oxidative damage to cells and creation of DNA adducts are the foremost among them. In this study, WR1065, active form of amifostine shows capacity to reduce both of these cisplatin effects in non-dividing differentiated neurons. For that reason, it would be prudent to reconsider amifostine as a candidate for new treatment formulations that would increase its local delivery to neurons to prevent CIPN.

## Electronic supplementary material

Below is the link to the electronic supplementary material.

Supplementary material 1 (DOCX 4314 KB)
