# Supplementary material for: Neuroprotective Role of Selected Antioxidant Agents in Preventing Cisplatin-Induced Damage of Human Neurons In Vitro
Source: Cell Mol Neurobiol. 2019 Mar 14;39(5):619–36. doi: 10.1007/s10571-019-00667-7 (PMC6535150; doi:10.1007/s10571-019-00667-7)
Supplement: Supplementary file 1 — Supplementary material 1 (DOCX 4314 KB) [file 10571_2019_667_MOESM1_ESM.docx]

Journal: Cellular and Molecular Neurobiology

**SUPPLEMENTAL DATA**

**Neuroprotective Role of Selected Antioxidant Agents in Preventing Cisplatin**

**Induced Damage of Human Neurons *in vitro***

Jelena Popović^#1,2^, Andrijana Klajn^#1^, Tatjana Paunesku^2^, Qing Ma^3^, Si Chen^4^, Barry Lai^4^, Milena Stevanović^1,5,6,*^, Gayle E Woloschak^2,*^

Corresponding authors:

Gayle E. Woloschak, Ph. D., Professor
Departments of Radiation Oncology, Radiology, and Cell and Molecular Biology
Robert H. Lurie Comprehensive Cancer Center
Feinberg School of Medicine
Northwestern University
300 E. Superior St., Tarry 4-760
Chicago, IL 60611

###### Milena Stevanovic, Academician, Professor

1. Institute of Molecular Genetics and Genetic Engineering,

University of Belgrade,Vojvode tepe 444a, Belgrade, 11010 Serbia

5. University of Belgrade, Faculty of Biology, Studentski trg 16, Belgrade, 11000, Serbia

6. Serbian Academy of Sciences and Arts, Knez Mihajlova 35, Belgrade, 11000, Serbia

**SUPPLEMENTAL DATA**

| Compound | Chemical shifts (eV) | XAS |
| --- | --- | --- |
| Cisplatin | 0.85 | EXAFS, XANES |
| Cisplatin+WR1065 | 1.00 | XANES |
| Cisplatin +Cells | 1.50 | XANES |
| Cisplatin+Cells+WR1065 | 1.60 | XANES |
| Cisplatin +DNA | 1.25 | EXAFS, XANES |
| Cisplatin +BSA | 1.40 | EXAFS, XANES |
| Cisplatin+DNA+WR1065 | 1.24 | EXAFS, XANES |
| Cisplatin+BSA+WR1065 | 1.55 | EXAFS, XANES |

**Supplemental Table 1. Samples used for Figure 8.** Samples were prepared on two occasions as detailed in methods. Second column shows chemical shifts (±0.04 eV) of the Pt L_III_ edges vs. Pt metal foil.

**
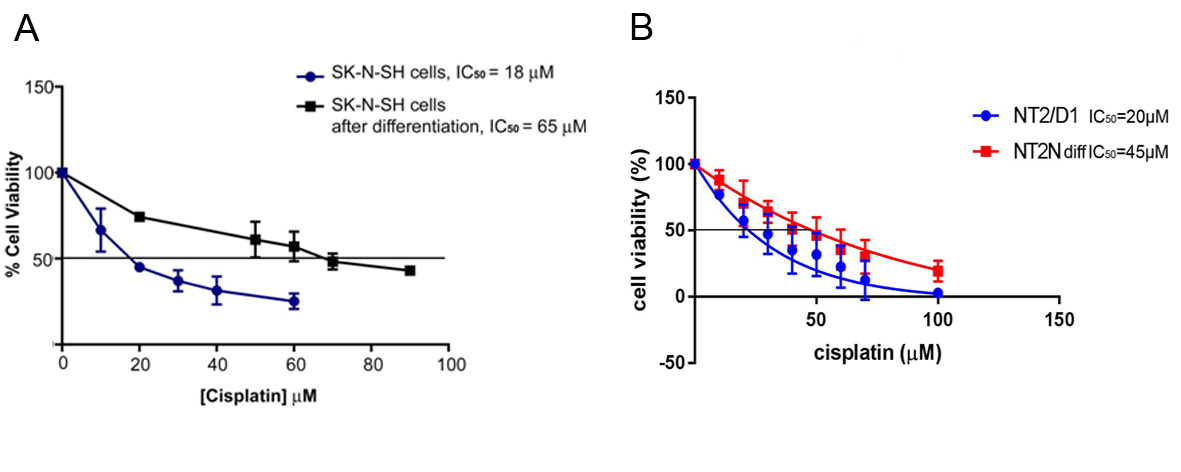
**

**Supplemental Figure 1. Effect of cisplatin on differentiating and proliferating neuroblastoma cells.** IC_50_ for undifferentiated (blue) and differentiated (black) SK-N-SH cells. Note the three fold higher cisplatin concentration needed to cause death of one half of the differentiated cells compared to undifferentiated cells.


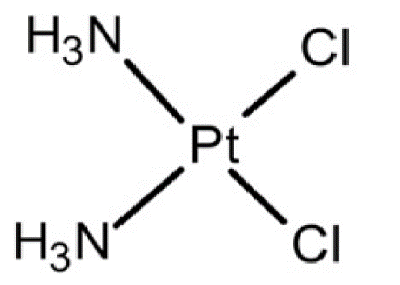
Cisplatin


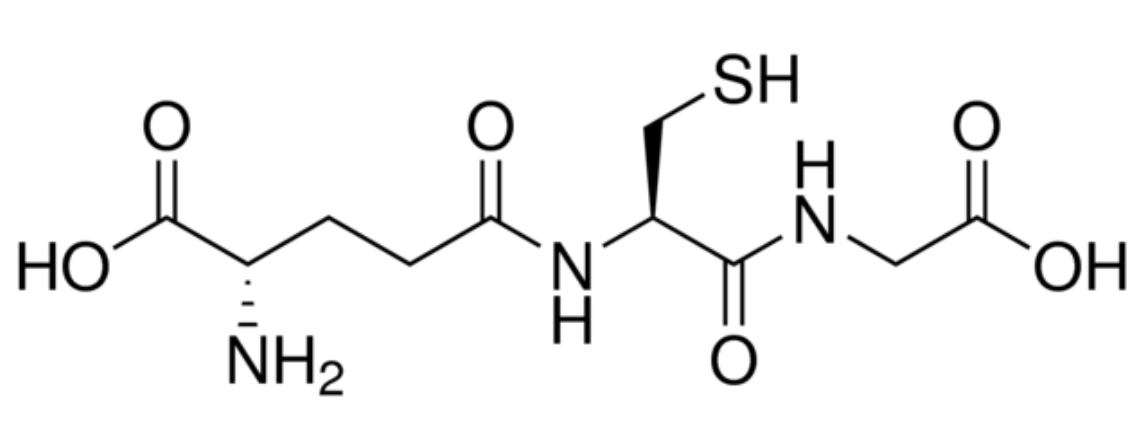


Glutathione


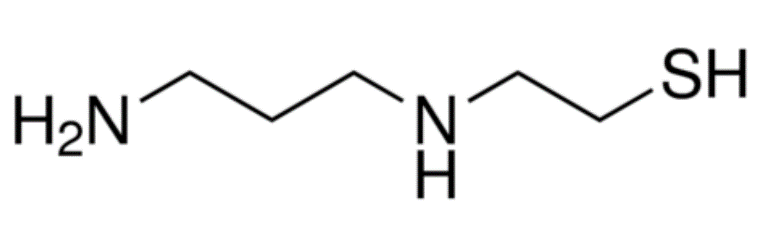
WR1065


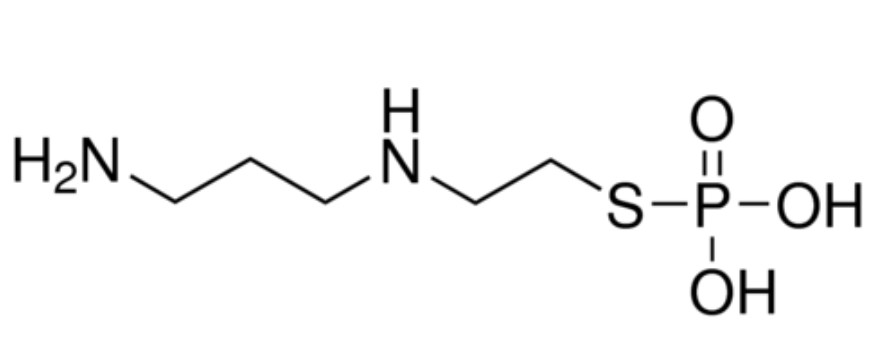


Amifostine


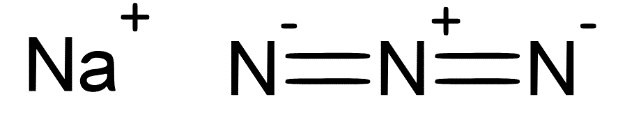
Sodium azide

**Supplemenmtal Figure 2**. Chemical formulae of cisplatin, intracellular thiol glutathione (γ-Glu-Cys-Gly), active thiol form (WR1065) of amifostine (C_5_H_14_N_2_S), amifostine (C_5_H_15_N_2_O_3_PS) and sodium azide (NaN_3_).

**
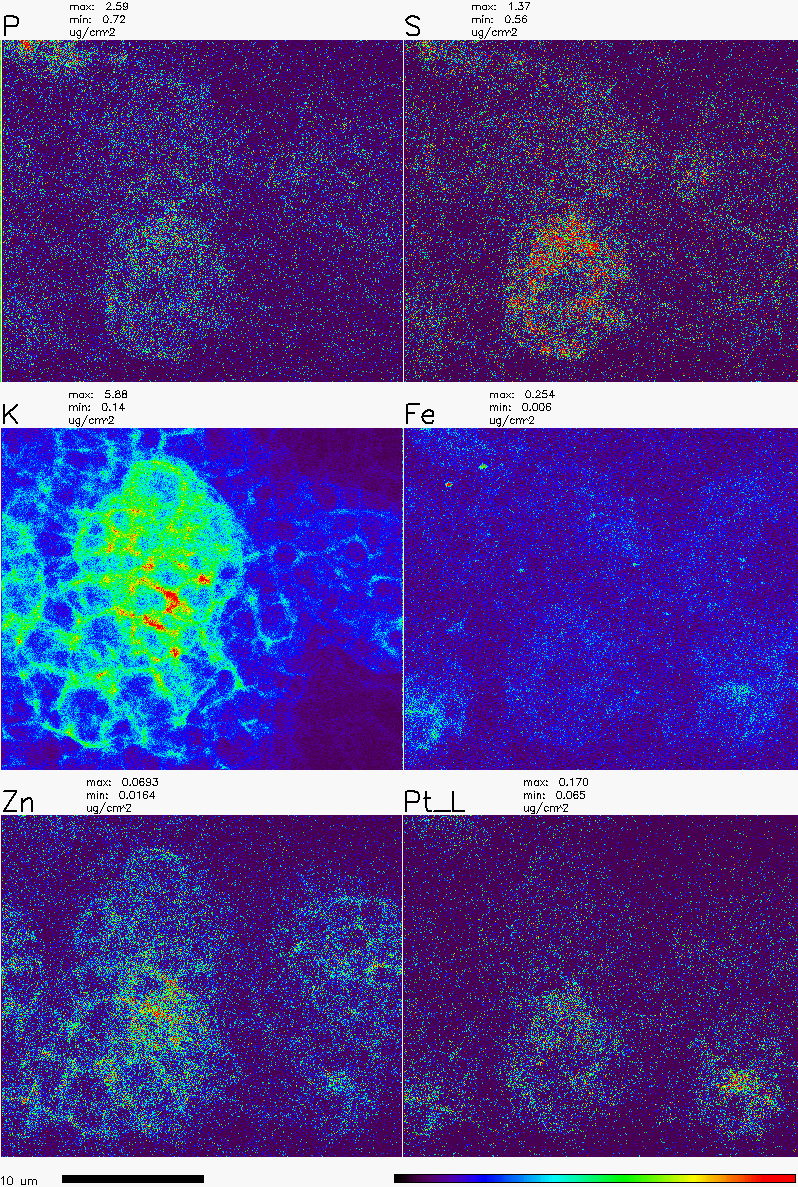
**

**Supplemental Figure 3. Detailed scan of a few NT2-N cells treated with cisplatin.** NT2-N cells were exposed to 100 μM cisplatin for 1 hour, washed with cisplatin free media and incubated without cisplatin for additional two hours prior to flash freezing in liquid ethane. Sample was maintained and imaged at cryogenic temperature. At the cisplatin concentration and incubation timepoint used, most of the cells are expected to be alive at the moment of freezing. Diminished capacity to retain high intracellular potassium concentration is an early sign of cell death; one such cell is present in this image in the lower right hand corner (pink arrowhead). At the same time this cell has the highest concentration of Pt. While this cell has similar Zn and Fe content as K high cells, K is completely absent from it. X-ray fluorescence microscopy was done at the Bionanoprobe instrument with 85 nm X-ray beam size in order to provide highest possible XFM resolution. At the same time, XFM done at the 2IDD beamline provided a higher sample throughput (Figures 6 and 7). Scale bar here is 10 microns, color bar indicates pixel concentrations for each element – from black (no signal) to red (highest signal for a given element). Concentration range per-pixel is indicated above each element’s map (e.g. Pt concentration goes from 0.085 μg/cm^2^ (dark blue pixels) to ≥ 0.17 μg/cm^2^ (red pixels).


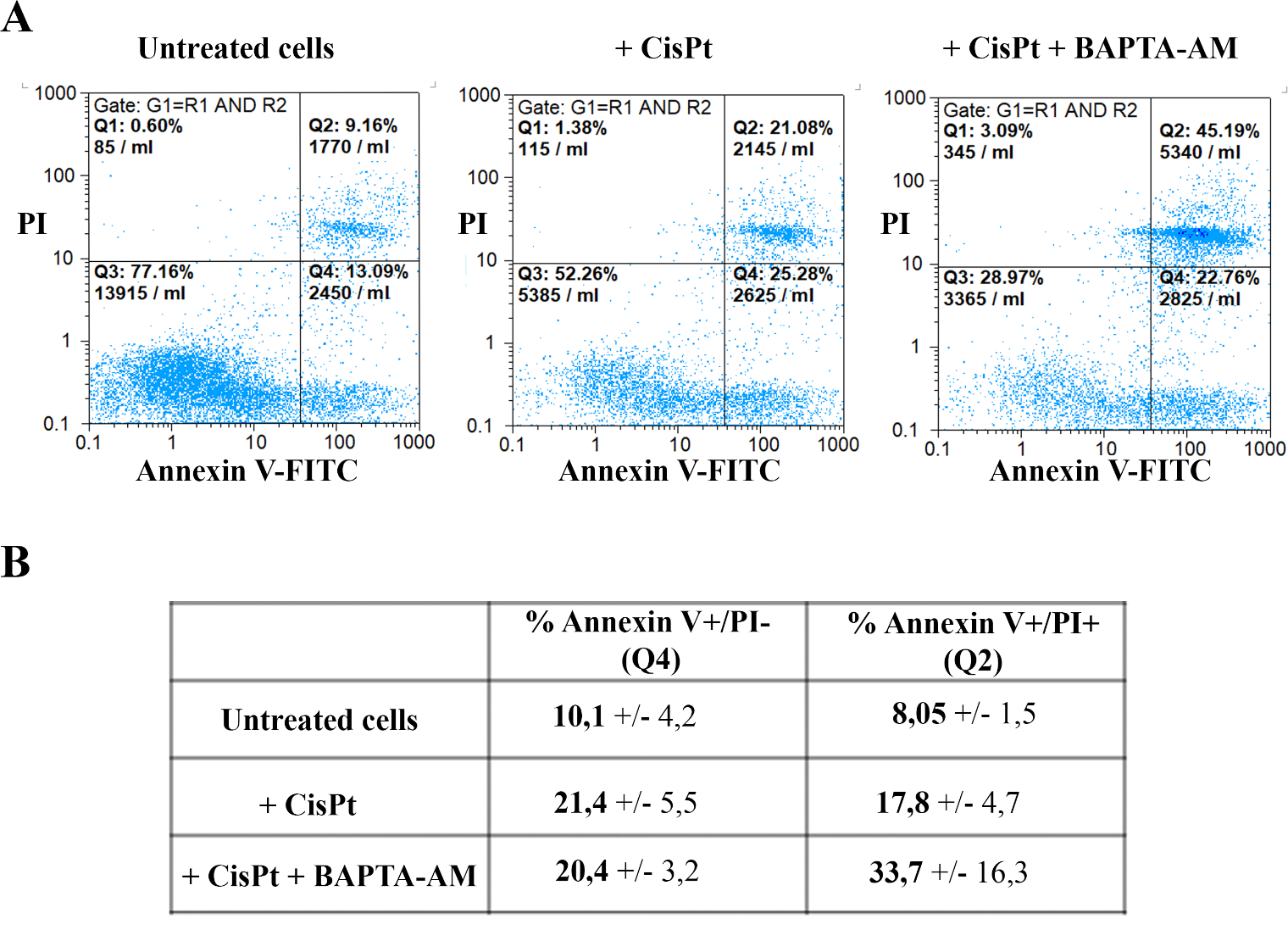


**Supplemental Figure 4.** A) NT2-N cells were exposed to cisplatin alone or in combination with BAPTA-AM (Sigma-Aldrich) at final concentrations: of 5 µM. Similar results were obtained with 1 µM (data not shown). At 24 h after treatment the cells were washed with cold PBS and resuspended in 1× Annexin binding buffer at a final number of 1 × 106 cells/ml. Five microliters of Annexin V (Annexin V, Alexa Fluor® 488 conjugate, Invitrogen™) and 5 μl of propidium iodide (PI—Invitrogen, 1mg/ml) were added. The cells were gently mixed, incubated for 15 minutes in the dark at RT and analyzed by CyFlow® Space Partec using the PartecFloMax® software (Partec GmbH, Münster, Germany). B) Summarized data of cells in early and late apoptosis are presented in the table as means of two independent experiments with standard deviation.
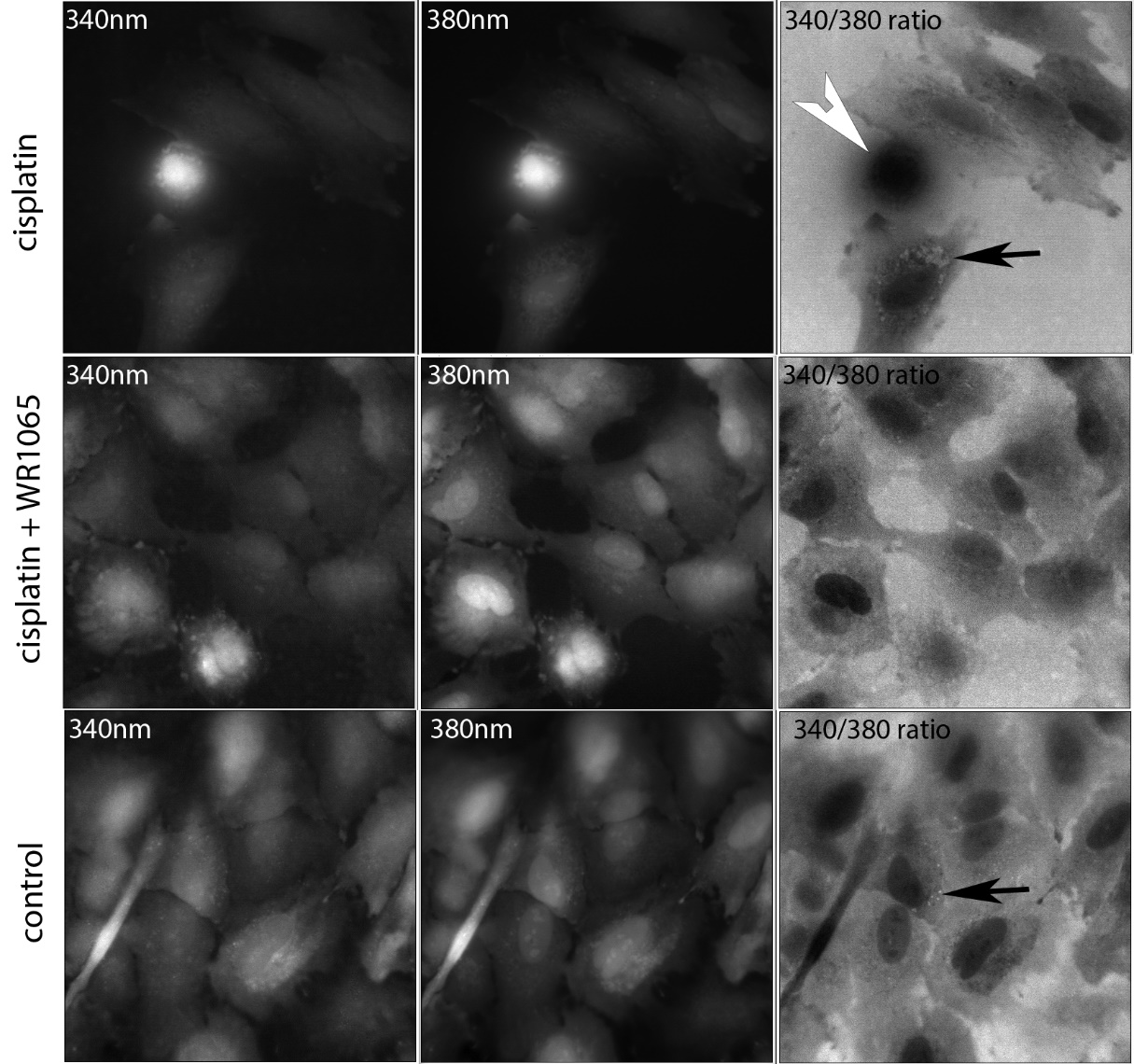


**Supplemental Figure 5**. SK-N-SH cells treated with cisplatin alone or in combination with WR1065 were incubated with FURA2 (Thermo Scientific), dye used for ratiometric imagining of calcium (Srikanth and Gwack 2013). Images obtained at 340nm and 380nm were divided using FIJI software and the resultant images (last column) showed calcium accumulation (black arrow) in untreated cells in small intracellular vesicles or in larger regions close to cell nucleus (in cisplatin treated cells). A single apoptotic cell - white arrowhead has a high concentration of metals (it is bright in both 340 and 380nm images), but the ratio of 340/380 nm images shows that this already apoptotic cell has a significant drop in Ca content. Imaging was done using confocal DSU Olympus microscope with 40x lens suitable for UV light wavelengths.

Srikanth S, Gwack Y. 2013. Measurement of intracellular Ca2+ concentration in single cells using ratiometric calcium dyes. Methods in molecular biology (Clifton, NJ). 963:3-14. eng.

**Supplemental Figure 6**. NaN_3_ and WR1065 modulate Pt spectra differently in mixtures of DNA and cisplatin. Note the changes in the peak height and shoulder height in the sample mixed with WR1065. In comparison, addition of NaN_3_ removed the shoulder and widened the peak of the Pt spectrum.
